# Continuous versus intermittent bolus infusion of calcium in preterm infants receiving total parenteral nutrition: a randomized blind clinical trial

**DOI:** 10.1186/s12887-023-04516-x

**Published:** 2024-01-12

**Authors:** Siamak Shiva, Khatereh Rezazadeh, Asmar Amiraslanzadeh, Bahareh Mehramuz, Sadollah Yeganehdoost, Majid Mahallei

**Affiliations:** 1 Pediatric Health Research Center, Tabriz University of Medical Sciences, Tabriz, Iran; 2 Department of Pathology, Faculty of Medicine, Tabriz University of Medical Sciences, Tabriz, Iran

**Keywords:** Preterm infant, Calcium, Alkaline phosphates, Metabolic bone disease

## Abstract

**Background:**

Premature neonates need adequate nutritional support to provide sufficient essential nutrients for optimal growth. Calcium (Ca) is one of the important nutrients in parental nutrition support of premature infants. This study aimed to compare the effect of continuous and intermittent bolus infusion of Ca on the incidence of metabolic bone disease (MBD) in preterm infants.

**Methods:**

This randomized double-blind clinical trial was conducted on ninety preterm infants in the NICU of Al-Zahra Hospital in Tabriz, Iran. The preterm infants were randomly allocated to either a continuous infusion group (received 4–5 ml/kg/day of Ca gluconate 10% by PN solution in a 24-h period) or an intermittent bolus administration group (received 1–2 ml/kg/day Ca gluconate 10% three to four times per day). Serial serum levels of Ca, phosphorous, alkaline phosphatase (ALP), vitamin D and parathyroid hormone (PTH) were assessed on the 7th day, 30th day and 45th day of life.

**Results:**

A total of 78 infants completed the study. The serum ALP level on the 45th day after birth was 753.28 ± 304.59 IU/L and 988.2 ± 341.3 IU/L in the continuous infusion and intermittent bolus administration groups, respectively (*P* < 0.05). MBD in preterm infants with ALP levels above 900 IU/L on the 45th day of life was significantly lower in the continuous infusion group than in the intermittent bolus administration group (*p* < 0.05). The mean serum levels of calcium, phosphorus, vitamin D and PTH in 45-day-old infants were not significantly different between the two groups.

**Conclusion:**

The MBD in preterm infants who received continuous infusion of Ca was lower than that in preterm infants who received intermittent bolus administration of Ca.

**Trial registration:**

The Iranian Registry of Clinical Trials (http://www.irct.ir) with the identification No. IRCT20210913052466N1.

## Introduction

In premature neonates, especially those with very low birth weight (< 1500 g), providing sufficient nutritional support is challenging and may need parenteral nutrition (PN) initiated as soon as possible after birth to decrease health disparities [1]. Inadequate nutritional support has been associated with short stature, poor neurodevelopmental outcomes, metabolic diseases, and postnatal growth restriction [2–10]. PN solutions consist of nutrients, electrolytes, minerals and vitamins, which are essential for the growth and metabolism of infants [11].

Calcium (Ca) and phosphorus (P) are essential elements of PN solutions of preterm infants, as both are needed for normal muscle function, energy production, bone health and skeletal mineralization. Providing adequate intake of Ca, P and vitamin D in PN solutions is necessary for skeletal growth and prevention of rickets, osteopenia or metabolic bone disease (MBD) of prematurity [12]. Children and infants, especially premature infants, require more Ca and P per kilogram of their weight to provide sufficient supplies for skeletal growth [13]. However, the supply of adequate amounts of Ca and P in PN solutions is mostly limited because of the lack of access to the central vein and solubility problems of Ca and P [14]. Most accumulation of Ca and P has occurred in the last 6 weeks of pregnancy. Therefore, preterm infants are at greater risk for MBD. It has been estimated that in the last trimester of pregnancy, the acquired Ca and P are approximately 120 to 150 mg/kg per day and 70 to 85 mg/kg per day, respectively [15].

It has been reported that intravenous infusion of Ca may cause important side effects such as skin rash, itching, pain and irritation at the injection site, seizure, bradycardia, cardiac arrhythmia, and skin necrosis resulting from subcutaneous deposition of Ca [12]. Furthermore, rapid administration of Ca is associated with vasodilation, hypotension, bradycardia, syncope, cardiac arrhythmias, and cardiac arrest. Administration by continuous infusion is more efficacious than intermittent bolus dosing due to less renal calcium loss [16].

When we have access to the peripherally inserted central catheter (PICC), calcium can be infused as bolus or continuous. But, when we only have access to peripheral vein, calcium can be infused just as bolus. Recently, it has been possible in neonatal intensive care units (NICUs) of our country the access to PICC, however, their usual method for calcium infusion is bolus method. Due to the urgent need for Ca in the PN of preterm infants to prevent related complications and the shortage of trained staff in NICUs for precise control of intravenous infusion of Ca, it is suggested that intravenous Ca can be administered as mixed with other PN solutions by continuous infusion via the central vein [12]. On the best of our knowledge, the effect of continuous administration of calcium compared to its bolus administration on the rate of MBD has not been studied in preterm infants. Therefore, we aimed to compare the effect of continuous and intermittent bolus infusion of calcium on the occurrence of MBD in preterm infants receiving total parenteral nutrition.

## Methods and materials

### Subjects

This study was carried out at the neonatal intensive care unit (NICU) of Al-Zahra Teaching Hospital, Tabriz, Iran, a university level III neonatal center in northwestern Iran, from September 2021 to November 2022. Ninety preterm newborn infants aged < 32 gestational weeks and/or with a birth weight < 1500 g who received total parenteral nutrition were included in our study. Those with severe asphyxia, chromosomal abnormalities, congenital metabolic abnormalities, malignancy, and maternal history of hyperparathyroidism and/or vitamin D abnormalities were excluded from this study.

### Sample size

The sample size was calculated based on differences between calcium levels in two groups of the study of Abdallah et al. [17], using the Pocock formula, with a confidence level of 95% and a power of 90%. At least 42 patients were needed for each intervention arm of the study. Anticipating a dropout rate of 10%, 45 infants were assigned to each group of the study.

### Study design

This randomized blind clinical trial was conducted in a 1:1 allocation ratio. The preterm infants were randomly allocated to two groups of continuous infusion and intermittent bolus calcium administration. The Random Allocation Software (RAS) were used for randomization allocation. The blocks were stratified by sex and age (block size = 4). The allocation was conducted by a statistician who was not involved in the study. All infants received PN according to the parenteral nutrition protocol used by the NICU of Al-Zahra Hospital. The continuous infusion group received 4–5 ml/kg/day (400–500 mg/kg/day) of calcium gluconate 10% by PN solution in a 24-h period, and the intermittent bolus administration group received 1–2 ml/kg/dose (100–200 mg/kg/dose) calcium gluconate 10% three to four times in the day (400–500 mg/kg/day). Moreover, all infants received phosphor at a Ca:P molar ratio of 1-1.3:1 in PN solution in 24 h.

PN admixtures were mixed under aseptic conditions in a clean room of the PN center of the hospital according to the center’s protocol by a pharmacy technician who was blind to the allocation sequence. To prevent Ca-P precipitation and reaction, first, phosphorous as sodium glycerophosphate (Glycophos 1 mmol/kg/day, Fresenius Kabi’s, USA) was added to a 10% dextrose solution, then amino acids and other nutrients were added, and finally, calcium as calcium gluconate (1-1.3 mmol/kg/day) was added. Then, PN solutions were sent to the NICU setting and infants randomly allocated to each group. The parents of infants were blind to the allocation treatment.

The demographic data including gestational age, sex, delivery type, birth weight, and Apgar score were recorded for all studied infants. Furthermore, duration of intravenous infusion of calcium, amount of infused gluconate calcium 10% and sodium glycerophosphate were reported. Time of initiation of breast milk and receiving of 100 ml/kg of enteral feeding were also evaluated.

Serum Ca, phosphorus, vitamin D, ALP, and PTH level were measured at 7th, 30th, and 45th day of birth of infants. The concentration of Ca, phosphorus and ALP level were measured using an automatic biochemical analyzer (Selectra, Netherlands). Serum level of vitamin D were assessed by ELISA method. The level of serum PTH was analyzed using electrochemiluminescence assay.

In this study, two criteria were applied to the diagnosis of osteopenia in all infants at 45 days of life. In the first criteria, an ALP level above 900 IU/L alone was used, and in the second criteria, an ALP level above 900 IU/L along with a serum phosphate level above 5.6 mg/dl (1.8 mmol/L) was used.

### Statistical analysis

The Kolmogorove-Smirnov goodness-of-fit was used for assessment of the normality of data distribution. The normal and non-normal distributed numeric variables were expressed as mean (standard deviation) and median (quartile 1, quartile 3), respectively. The categorical data were also shown as number (percent). The possible difference between intervention groups at baseline were evaluated by chi-square test and independent sample t-test. The within-group differences were analyzed using repeated measures. For identify the differences in response to the treatment, analysis of covariance (ANCOVA adjusted for 30th day values, vitamin D and phosphorous or calcium) was performed. The data analysis were done on an intention-to-treat basis by the SPSS version 22, software package (SPSS Inc, Chicago, IL). *P* values less than 0.05 were considered statistically significant.

## Results

Out of 93 preterm infants who enrolled to participate in the study, 3 infants did not meet the inclusion criteria because of severe asphyxia (*n* = 2) and congenital metabolic abnormalities (*n* = 1). Among 90 preterm infants who were included in the study, five and seven subjects were lost to follow-up in the continuous and intermittent bolus administration groups, respectively. The cause of the death in both groups was sever prematurity (gestational age < 27 weeks) and complication of respiratory distress syndrome (RDS) in first three days of life.

**Fig. 1:**
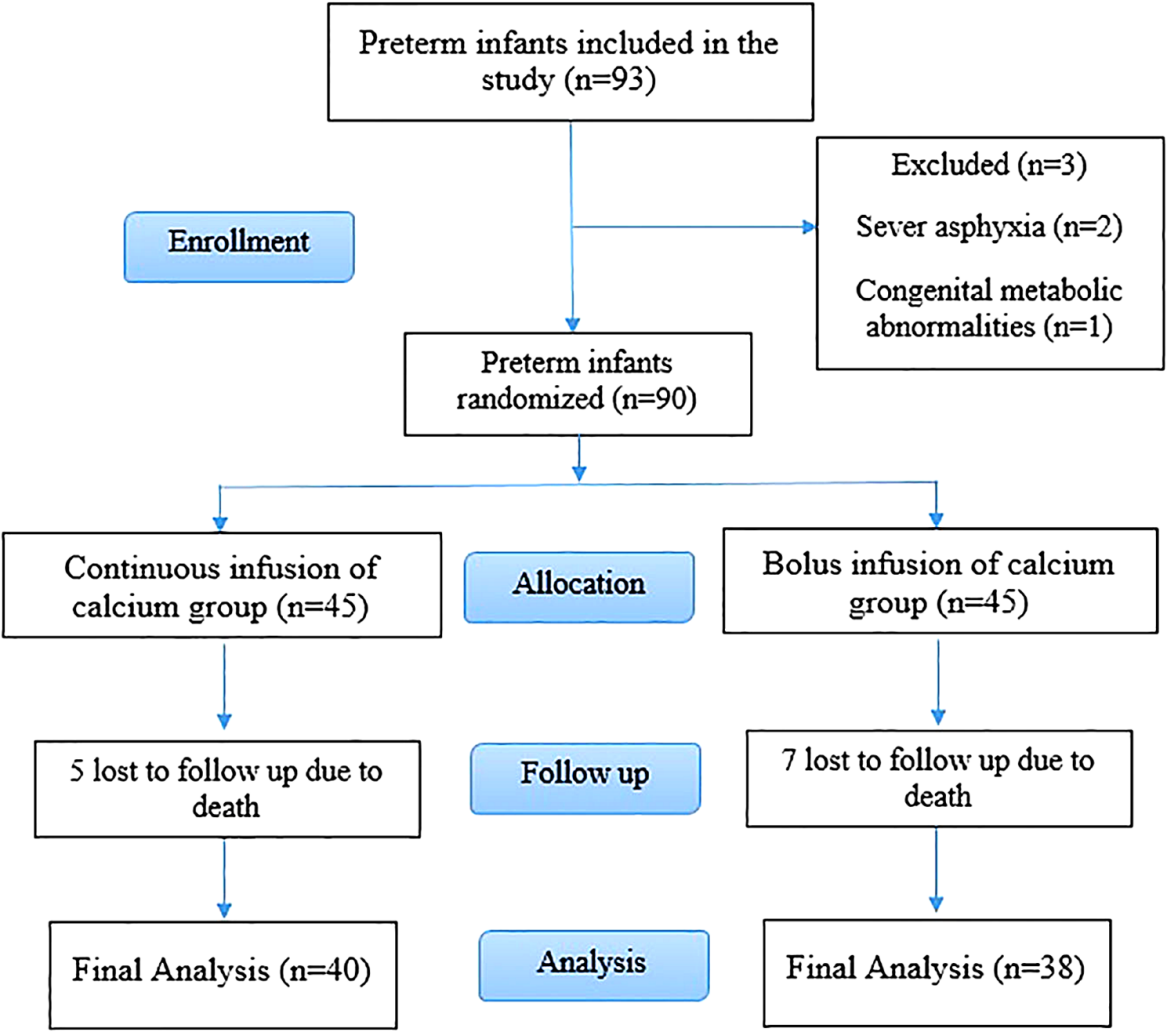
Flow diagram of study process

Therefore, 78 subjects completed the study (continuous infusion group = 40, intermittent bolus administration group = 38) (Fig. 1).

There were no reports of adverse effects of Ca infusion in either of the groups during the trial. The preterm infants who received Ca as continuous infusion and intermittent bolus administration were homogeneous for demographic characteristics, including gestational age, sex, delivery type (vaginal delivery and cesarean delivery), birth weight, and Apgar score 1 and 5 min (Table 1). No significant differences between the two groups of subjects were found in the duration of intravenous infusion of calcium. Infants in the intermittent bolus administration group received significantly less calcium than infants in the continuous infusion group (*P* < 0.001). However, between-group analysis showed no significant differences in serum calcium levels at 45 days (Table 2).

**Table 1:** Baseline characteristics of preterm infants

variables		Continuous infusion group (*n* = 40)	Bolus infusion group (*n* = 38)	*P*-value^†^
Gestational age (week)^$^		28.7 (2.3)	29.1 (1.8)	0.399
Sex ^#^				0.850
	Boy	16 (40.0%)	16 (42.1%)	
	Girl	24 (60.0%)	22 (57.9%)	
Delivery type ^#^				0.117
	Vaginal delivery	11 (27.5%)	5 (13.2%)	
	Cesarean delivery	29 (72.5%)	33 (86.8%)	
Birth weight (g)^$^		1085.2 (193.2)	1194.6 (176.8)	0.052
Apgar score 1^$^		5.63 (1.7)	5.1 (2.0)	0.239
Apgar score 5^$^		7.63 (1.7)	7.1 (1.3)	0.137

^$^ Mean (SD)

^#^ Number (%)

^†^ Independent t-test for numeric variables and Pearson Chi-Square test for categorical variables

**Table 2:** Time and amount of parenteral nutrition, enteral nutrition and breast milk in preterm infants who received continuous infusion of calcium and those received bolus infusion of calcium

variables	Continuous infusion group (*n* = 40)	Bolus infusion group (*n* = 38)	*P*-value
Duration of intravenous infusion of calcium (days) ^$^	17.5 (10.0)	19.8 (9.3)	0.295^†^
Amount of infused gluconate calcium 10% (ml/kg/day) ^$^	4.0 (0.4)	3.3 (0.4)	< 0.001^†^
Amount of sodium glycerophosphate (ml/kg/day) ^$^	1.0 (0.1)	0.9 (0.0)	0.498^†^
Start of breast milk (days) ^#^	3 (1–7)	2 (1.0-3.2)	0.130*
Reach to 100 ml/kg of enteral feeding (days) ^$^	24.3 (12.0)	17.6 (9.6)	0.008^†^

^$^ Mean (SD)

^#^ Median (quartile 1, quartile 3)

^†^ Independent t-test

^*^ Mann-Whitney U test

Both groups of infants received the same amount of sodium glycerophosphate. The mean serum phosphorus level was lower than 5.6 mg/dl at 45 days in both groups, and there was no significant difference between the two groups in the serum phosphorus levels at that time. The time of the start of the breast milk was similar in the two groups of study infants. Infants in the intermittent bolus administration group reached 100 ml/kg of enteral feeding earlier than infants in the continuous infusion group (*p* = 0.008) (Table 2).

**Fig. 2:**
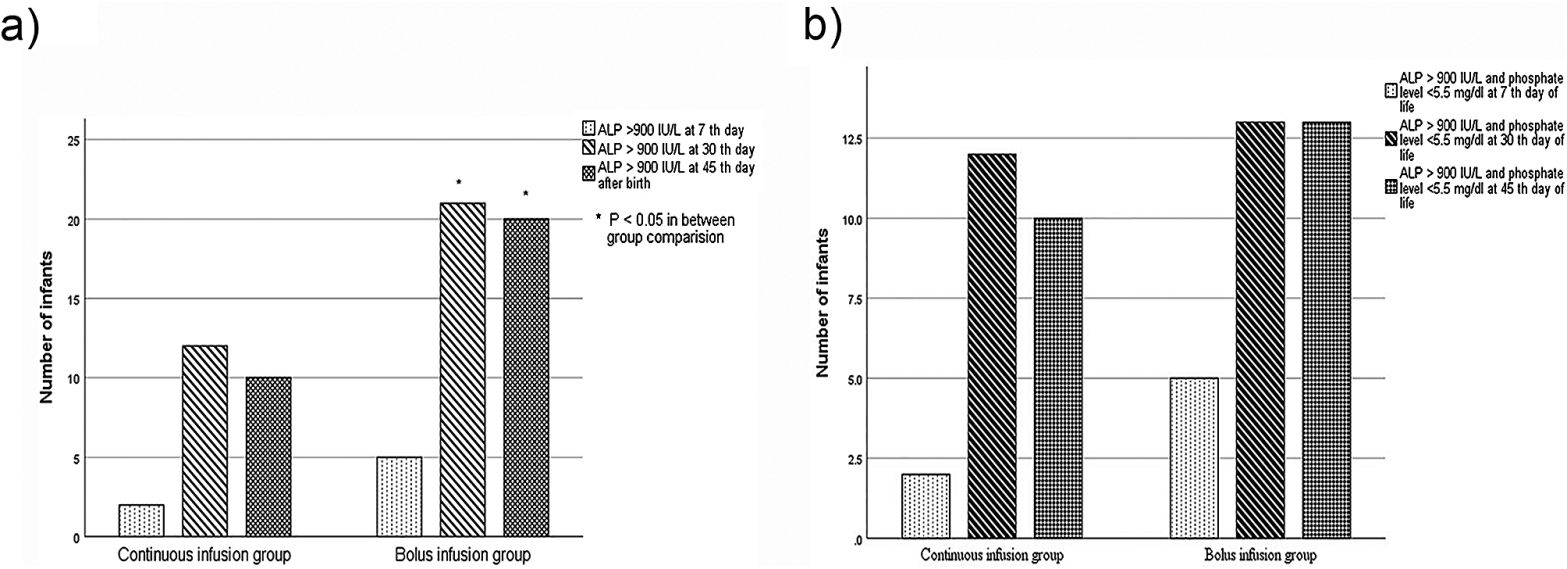
Number of infants with **(a)** ALP level above 900 IU/L and **(b)** ALP level above 900 IU/L along with phosphate level < 5.5 mg/dl at 7th, 30th and 45th day after birth in each group of intervention

The serum ALP level on the 45th day after birth was 753.28 ± 304.59 IU/L in the continuous infusion group and 988.2 ± 341.3 IU/L in the intermittent bolus administration group (*P* = 0.002). Moreover, ALP levels increased significantly from the 7th day to 30th day and from the 7th day to 45th day after birth in both groups of infants. ALP levels above 900 IU/L were reported in 12 infants in the continuous infusion group and 21 infants in the intermittent bolus administration group on the 30th day of life (p 0.05). On the 45th day of life of infants, ALP levels above 900 IU/L were observed in 10 and 20 infants in the continuous and intermittent bolus administration groups, respectively (p 0.05). The number of preterm infants with ALP 900 IU/L along with phosphorous 5.6 mg/dl was not different between the two groups (Fig. 2).

**Fig. 3:**
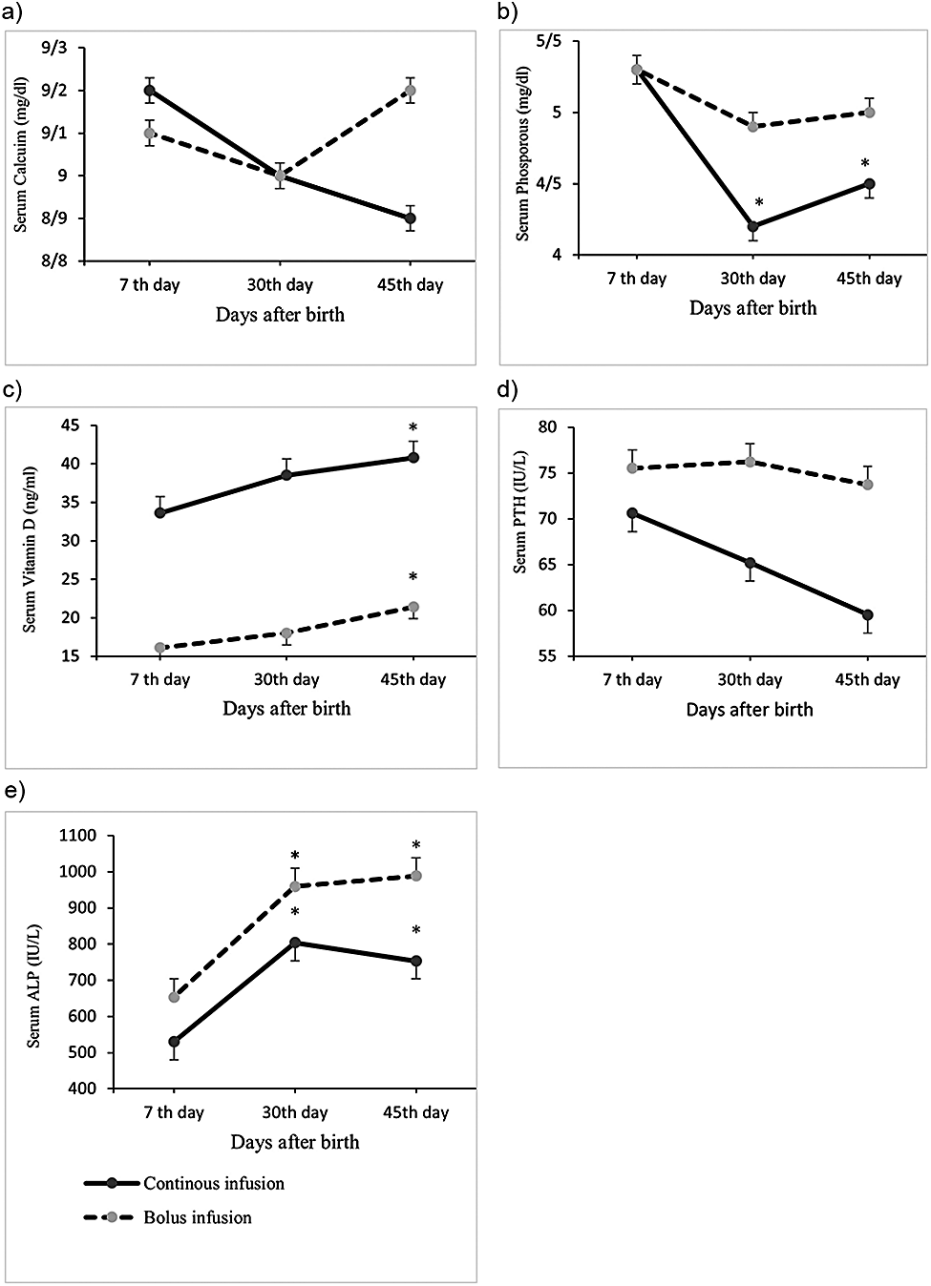
Mean values of biochemical markers at 7th, 30th and 45th day after birth in preterm infants with continuous infusion of calcium and with bolus infusion of calcium. ^*^P < 0.05 in repeated measure in each group

Vitamin D levels increased significantly from 7 days to 45 days postnatal age in both groups of infants. Vitamin D levels were significantly higher in the continuous infusion group than in the intermittent bolus administration group on the 7th and 30th days (*p* < 0.001), but these differences were not significant on the 45th day of life after adjustment for confounding factors, including vitamin D levels on the 30th day and calcium and phosphorus levels. PTH levels were not changed significantly in infants in both group, and no significant between-group differences were found in PTH levels. Finally, the mean serum levels of calcium, phosphorus, vitamin D and PTH at 45 days old were not significantly different between the two groups (Table 3; Fig. 3).

**Table 3:** Comparison of biochemical markers between the study groups at 7th day, 30th day and 45th day after birth of preterm infants

Variable	Continuous infusion group (*n* = 40)	Bolus infusion group (*n* = 38)	*P*-value^†^
Serum calcium (mg/dl)			
7th day	9.2 (1.0)^a^	9.1 (0.8)	0.638^£^
30th day	9.0 (0.7)	9.0 (0.6)	0.888^£^
45th day	8.97 (0.6)	9.2 (0.6)	0.064^†^
Serum phosphorus (mg/dl)			
7th day	5.3 (1.2)	5.3 (1.1)	0.973^£^
30th day	4.2 (0.8)	4.9 (0.8)	0.000^£^
45th day	4.5 (0.9)	5.0 (0.7)	0.227^†^
Vitamin D (ng/ml)			
7th day	33.6 (15.3)	16.1 (10.2)	0.000^£^
30th day	38.5 (20.4)	18.0 (9.4)	0.000^£^
45th day	40.8 (19.6)	21.4 (11.0)	0.111^†^
ALP (IU/L)			
7th day	529.9 (221.0)	653.4 (198.3)	0.011^£^
30th day	803.7 (340.5)	959.2 (343.9)	0.048^£^
45th day	753.2 (304.5)	988.2 (341.3)	0.031^†^
PTH (IU/L)			
7th day	70.6 (58.6)	75.5 (55.2)	0.701^£^
30th day	65.2 (67.3)	76.2 (60.7)	0.453^£^
45th day	59.5 (57.5)	73.7 (52.9)	0.878^†^

MD: Mean Difference; ALP: Alkaline Phosphates; PTH: Parathyroid Hormone

^a^ Mean (SD). *P*-values of statistical significance (*p* < 0.05) are presented in bold

^†^ Analysis of covariance (adjusted for 30th day values, vitamin D and phosphorous or calcium)

^£^ Independent t-test

## Discussion

The present study evaluated the effect of continuous versus intermittent bolus infusion of Ca on the probability of incidence of MBD of prematurity based on ALP level and other biomarkers in preterm neonates with gestational age < 32 weeks and/or birth weight < 1500 g who received total PN after birth. Although it has been demonstrated that Ca is an essential micronutrient in PN solutions, the best method for parenteral Ca infusion has not been studied. This study indicated that the incidence of MBD was significantly lower in infants of the continuous infusion group compared to the intermittent bolus infusion group.

In the present study, based on the ALP level > 900 IU/L, the incidence of MBD in infants were 9% and 38.5% at 7th day and 45th day after birth, respectively. MBD is reported in 16–40% of very low birth weight (< 1500 g) and extremely low birth weight (< 1000 g) infants [18]. MBD in premature infants is caused by insufficient mineralization of bone matrix. The peak incidence of MBD is at 4–8 weeks after birth [19]. As in a normal pregnancy, two-thirds of the accumulation of calcium and phosphorous in the fetus is accrued during the last trimester of pregnancy, and prematurity increases the risk of diminished bone mineral stores and the incidence of MBD [20]. Other factors, including bronchopulmonary dysplasia, long-term total PN, immobility, intake of corticosteroids and diuretics and inadequate intake of Ca, phosphorous and vitamin D, may contribute to MBD incidence [21–23].

The diagnostic gold standard for MBD detection is dual energy X-ray absorptiometry (DEXA), which can identify bone mineralization status. However, this diagnostic method has limited use because of high costs, unavailability and logistic requirements [24]. Furthermore, X-ray radiography in the skeletal system cannot easily detect MBD until the bone mineral content is reduced by 20–30% [25]. Biochemical markers of bone mineralization, such as serum ALP and, to a lesser extent, serum Ca, phosphorous and 25-hydroxy vitamin D, can be used for the detection of MBD in preterm infants [26–28].

In this study, serum ALP and phosphorous levels were used for MBD diagnosis. Based on ALP > 900 IU/L at 45 days postnatal age, 52.6% of preterm infants in the intermittent bolus calcium administration group were diagnosed with MBD of prematurity, whereas in those of the continuous infusion group only 25% had MBD, and these differences between the two groups were statistically significant (*P* < 0.05). However, when ALP > 900 IU/L along with phosphorous < 5.6 mg/dl were applied as diagnostic criteria for MBD, the incidence of MBD was 34.2% in the intermittent bolus administration group and 25% in the continuous infusion group which were not significantly different between the two groups. In addition, there was an increasing trend in the serial measurements of ALP levels on days 7 and 30 in both groups. The mean serum level of ALP was significantly lower in infants of the continuous infusion group compared to the intermittent bolus administration group on days 7 and 30. (*P* < 0.05). However, the ALP level decreased in the continuous infusion group, but increased in the intermittent bolus administration group between 30th and 45th days of postnatal age and the differences between groups were statistically significant even after adjustment for baseline values and other confounding factors (*P* < 0.05). Finally, in the present study, MBD (based on ALP > 900 IU/L) was detected in 10 infants (25%) in the continuous infusion group and in 20 infants (52.6%) in the intermittent bolus administration group (*P* < 0.05).

Several studies have suggested serum ALP levels as a diagnostic biomarker for MBD of prematurity. It is shown that serum ALP is a reliable biomarker for assessing bone metabolism and increased levels of ALP reflect elevated bone turnover and disturbance of the mineralization process [28, 29]. It has also been reported that serial measurements of ALP weekly or biweekly may increase the accuracy of the diagnosis of MBD [23, 29]. Nevertheless, variable cutoff values of ALP were applied in studies for screening MBD of prematurity. Some studies selected ALP > 500 IU/L as the threshold value for predicting MBD, which varies their sensitivity (from 76.1% [22] to 100% [17]) and specificity (from 66.6% [22] to 80.7% [17]) [17, 22, 30], whereas Hung et al. [28] reported that ALP > 700 IU/L at the 3rd week after birth could predict MBD with 73% sensitivity and 74% specificity. Moreover, Backstrom et al. found that an ALP level > 900 IU/L is predictive of MBD with a sensitivity of 88% and a specificity of 71% [31]. The different cutoff values of ALP in studies to predict MBD may be attributed to the subjects of the studies. As in Backstrom et al.‘s study, neonates with median birth weight of 1500 g (range 735–2250) and a median gestational age of 30 weeks (range 24.7–33.0 weeks) were chosen [31], while in Viswanathan et al.‘s study, the subjects were extremely low birth weight (< 1000 g) with a gestational age < 30 weeks [22]. Currently, there is no constant cutoff of serum ALP to distinguish MBD of prematurity. However, as shown by Backstrom et al., a combination of ALP level > 900 IU/L and serum inorganic phosphate level < 1.8 mmol/L (5.6 mg/dl) predict low bone mineral density by 100% sensitivity and 70% specificity compared to DEXA results [31]. Therefore, assessment of serum ALP and phosphate levels could be used to screen preterm infants who are at risk for MBD [26].

In our study, the ALP serum level on the 45th day after birth was 988.2 ± 341.3 IU/L in the intermittent bolus administration group and 753.28 ± 304.59 IU/L in the continuous infusion group (*P* < 0.05). Differences in MBD incidence between continuous infusion and intermittent bolus administration of calcium in preterm infants may be the result of stable serum levels of calcium when continuous infusion was used. Although PN administration via central venous circulation is preferred for long-term usage, the risk of sepsis and problems in the maintenance of surgically inserted central lines are limiting factors. Thus, peripherally inserted central catheter (PICC) lines are the best alternative route for PN delivery [32]. Based on the pediatric parenteral nutrition guidelines, when PN is administered via the central line, Ca and phosphate can be mixed in PN solutions [32–34]. However, when PN is given via the peripheral vein, osmolality (less than 600 mOsml/L) and the concentration of nutrients in the admixtures are restricted because of adverse effects such as tissue necrosis and phlebitis [35]. Thus, in peripheral access, phosphate as sodium glycerophosphate is added to the PN solutions, and Ca is infused separately.

The present study showed that the mean phosphorus serum level in both groups was lower than 5.6 mg/dl. However, the number of patients who had a serum phosphorus level lower than 5.6 mg/dl was significantly higher in the intermittent bolus administration group (13 people) than in the continuous infusion group (3 people) (*P* = 0.003). According to the available studies, it should be noted that although the serum phosphate concentration was lower in the osteopenia group in most studies, the prediction accuracy with this method was significantly lower than that with ALP [28]. Catache and Leone also proved that serum phosphate levels are not good markers for the early detection of mineral deficiencies. They argued that while a decrease in phosphate concentration may be responsible for osteopenia, it may not adequately reflect a state of bone mineral deficiency [36].

In the present study, intravenous calcium was prescribed as a part of intravenous nutrition for both study groups. The starting dose in both groups was 4–5 ml/kg/d, but according to the final data of the study, it was found that the mean intravenous calcium administration in the bolus group was significantly lower than that in the continuous infusion group (Table 2). This difference can be result of treating doctors in bolus infusion method usually reduce the calcium infused because of possible risks of subcutaneous deposition of calcium. So in our results, the mean of infused calcium was lower in bolus group than continuous group. However, duration of calcium infusion was not significantly different between two groups (Table 2). Moreover, there was no difference in the serum calcium level (at 7, 30 and 45th day) between two groups (*p* < 0.05) (Table 3). Whilst in bolus group, ALP level (at 7, 30 and 45th day) was significantly higher than continuous group (*p* < 0.05) (Table 3). These results were similar to the results of previous studies such as the study by Hung et al., who reported similar levels of calcium concentration in osteopenic preterm infants (less than 34 weeks of gestation) compared to those with no evidence of osteopenia [28]. Additionally, this was in agreement with the study of Abdallah et al. [17] and the study of So and Ng [37], who reported normal levels of calcium in osteopenic infants. Serum calcium levels are usually normal in infants with MBD due to the rapid response of calcium-sensing receptors in the parathyroid glands, and therefore, serum calcium is not a sensitive marker in MBD screening [19]. In addition, the serum calcium level may be affected by other disorders such as phosphate depletion and hypophosphatasia [26]. Recently, there has been interest in PTH and 25-hydroxyvitamin D as additional markers used to assess bone health status [19]. The AAP published a statement in 2013 recommending a target calcidiol level of more than 20 ng/mL [38]. Nevertheless, Moreira et al. found that the level of 25-hydroxyvitamin D in infants is often normal in infants with MBD [39]. The results of our study also showed that the serum levels of vitamin D and PTH at 45 days were not significantly different between the two groups (Table 3).

Generally, in most NICU settings in Iran, Ca is usually infused via PICC or peripheral access as intermittent bolus administration which may be one of the reasons for the high incidence of prematurity MBD in our nation [40]. In our study, continuous infusion of Ca via PICCs showed a lower incidence of MBD than intermittent bolus administration of Ca without possible adverse effects as the reliable available guidelines preferred the continuous intravenous infusion rather than the intermittent bolus infusion [32–34].

## Limitations

This study had some limitations such as the small number of infants, single-center NICU, relatively short follow up time and only measurement of biomarkers of MBD. Radiography and DEXA assessments were not used in the study because of funding constraints and logistical problems.

## Conclusion

The incidence of MBD was lower in preterm infants who received continuous infusion of Ca than in those who received intermittent bolus doses of Ca, when the ALP level was used as a diagnostic criterion for MBD detection. However, when ALP and phosphorus levels were used to diagnose MBD, although MBD was still higher in the intermittent bolus administration group, no significant difference was found between the two groups of patients.

## Data Availability

The datasets generated and/or analyzed during the current study are not publicly available due to the institution’s policy, but are available from the corresponding author upon reasonable request.
